# Factors associated with documented distress during venipuncture in routine health checkups

**DOI:** 10.3389/fpsyt.2026.1866329

**Published:** 2026-08-12

**Authors:** Yin Yuan, Yu-Chen Yen, Wei-Min Chu, Shih-Yi Lin, Chiann-Yi Hsu, Shu-Hui Yang, Cheng-Fu Lin

**Affiliations:** 1 Department of Nursing, Taichung Veterans General Hospital, Taichung, Taiwan; 2 Health Management Center, Taichung Veterans General Hospital, Taichung, Taiwan; 3 Department of Post-Baccalaureate Medicine, College of Medicine, National Chung Hsing University, Taichung, Taiwan; 4 Geriatrics and Gerontology Research Center, College of Medicine, National Chung Hsing University, Taichung, Taiwan; 5 Department of Family Medicine, Taichung Veterans General Hospital, Taichung, Taiwan; 6 School of Medicine, National Yang Ming Chiao Tung University, Taipei, Taiwan; 7 Center for Geriatrics and Gerontology, Taichung Veterans General Hospital, Taichung, Taiwan; 8 Biostatistics Group, Department of Medical Research, Taichung Veterans General Hospital, Taichung, Taiwan

**Keywords:** blood-drawing procedures, health examinations, needle fear, preventive healthcare, vasovagal response, venipuncture

## Abstract

**Purpose:**

Blood-drawing procedures are among the most commonly performed medical interventions in clinical practice and are routinely conducted during preventive health examinations. However, needle-related procedures may provoke fear and anxiety in some individuals and may influence healthcare experiences and engagement. Despite extensive research on needle fear in vaccination and pediatric contexts, limited attention has been paid to venipuncture-related distress during routine health examinations.

**Patients and methods:**

A retrospective cross-sectional study was conducted using health examination records from a tertiary medical center in Taiwan. Adults who underwent routine health examinations between January 1, 2023, and May 31, 2024 were eligible. Individuals younger than 18 years, those who did not undergo venipuncture, and records with incomplete data were excluded. Documented behavioral and physiological responses during venipuncture were used as proxy indicators of venipuncture-related distress. Bivariate analyses and multivariable logistic regression models were performed to evaluate associated factors.

**Results:**

Among 15,932 participants included in the final analysis, 162 individuals (1.02%) had documented behavioral or physiological distress during venipuncture. Participants with documented distress were significantly younger and had lower systolic blood pressure. In multivariable analysis, younger age (adjusted odds ratio (OR) = 0.96, 95% CI: 0.94–0.98), lower systolic blood pressure (adjusted OR = 0.98, 95% CI: 0.97–0.99), and colonoscopy (adjusted OR = 3.49, 95% CI: 2.01–6.06) were associated with documented distress.

**Conclusion:**

Approximately 1% of adults undergoing routine health examinations had observable venipuncture-related distress documented in routine nursing records. Recognition of needle-related distress during venipuncture may facilitate earlier identification of affected individuals and support strategies to improve patient experience during preventive healthcare.

## 1 Introduction

Blood-drawing procedures represent one of the most frequently performed medical interventions in clinical practice and are routinely conducted during preventive health examinations, diagnostic assessments, and chronic disease monitoring. The hypodermic needle remains an indispensable medical tool for venipuncture, vaccination, and medication administration, with billions of injections administered worldwide each year (1). Despite their essential role in modern healthcare, needle-related procedures are frequently associated with fear, anxiety, and psychological distress among both pediatric and adult populations (2, 3). These emotional responses may compromise patient comfort and may also reduce willingness to participate in routine medical care or preventive health services (1, 4).

Needle phobia is categorized as a subtype of blood-injection-injury (BII) phobia within the diagnostic and statistical manual of mental disorders and is characterized by an excessive and persistent fear of medical procedures involving needles that leads to avoidance behavior (5–7). Epidemiological investigations have suggested that BII phobia represents a distinct subtype of specific phobia with unique clinical and physiological characteristics compared with other phobic disorders (5, 8). Unlike most other specific phobias, BII phobia is associated with a characteristic diphasic vasovagal response consisting of an initial sympathetic activation followed by parasympathetic overcompensation, which may result in hypotension, dizziness, or syncope (7, 9, 10). In addition to cardiovascular responses, respiratory dysregulation, including hyperventilation-induced hypocapnia, has been observed during exposure to blood or injection stimuli and may contribute to the exacerbation of symptoms in susceptible individuals (9, 11).

Beyond physiological mechanisms, psychological processes have also been proposed to contribute to the development and maintenance of needle-related fear. Cognitive biases, including attentional vigilance toward threat-related stimuli and expectancy distortions, have been described in individuals with BII phobia and may contribute to anticipatory anxiety and avoidance behaviors (12). These cognitive processes may amplify perceived procedural threat and increase subjective distress when individuals are confronted with needle-related medical procedures (12).

Epidemiological evidence indicates that needle fear exists along a continuum ranging from mild discomfort to clinically significant phobia. Systematic review evidence suggests that a substantial proportion of adults report fear of needles, whereas clinically defined needle phobia affects a smaller proportion of individuals, typically between 1% and 4% (1). Broader estimates of injection-related distress have been reported in surveys relying on self-reported fear scales or symptom-based questionnaires (13–15). Fear of needles is particularly common among children and adolescents and tends to decrease with increasing age (3, 16). Certain populations may also exhibit heightened procedural anxiety. For example, individuals with neurodevelopmental disorders, including autism spectrum disorder, may experience increased distress during needle-related procedures and may require specialized management approaches (17, 18).

Clinically, unmanaged needle fear represents a substantial barrier to healthcare engagement. Needle-related anxiety has been associated with avoidance of vaccination, blood donation, and routine medical procedures (1, 4, 16). Among individuals with chronic diseases requiring repeated injections or blood tests, such avoidance behaviors may negatively affect treatment adherence and long-term disease management (4). In more severe cases, needle phobia may be accompanied by vasovagal syncope and significant physiological distress, which may complicate clinical procedures and create additional challenges for healthcare providers (19, 20).

Several evidence-based strategies have been proposed to mitigate needle-related fear and procedural distress. Psychological interventions such as exposure-based therapy, applied muscle tension techniques, and cognitive-behavioral coping strategies have demonstrated effectiveness in reducing needle-related anxiety and improving tolerance of medical procedures (21–23). Emerging technologies, including virtual reality-assisted exposure therapy and physiological monitoring methods designed to anticipate vasovagal responses, have also shown promising preliminary findings (24, 25).

Although needle fear has been extensively investigated in vaccination settings and pediatric healthcare contexts, relatively limited research has focused on blood-drawing procedures conducted during routine preventive health examinations. Venipuncture represents one of the most frequently performed medical procedures during health checkups and may serve as a trigger for anxiety or vasovagal reactions in susceptible individuals. Accordingly, the present study aimed to estimate the proportion of adults with behaviorally documented venipuncture-related distress during routine health examinations and to identify demographic and clinical factors associated with such documentation.

## 2 Materials and methods

### 2.1 Study design and setting

A retrospective cross-sectional study was conducted using routinely collected health examination records from the health examination center of a large tertiary medical center in Taiwan. The center provides standardized health screening services for adult populations, including laboratory testing, imaging studies, and endoscopic examinations as part of routine preventive health checkups. The study was conducted and reported in accordance with the strengthening the reporting of observational studies in epidemiology guidelines for observational studies (26).

### 2.2 Study population

**Figure 1:**
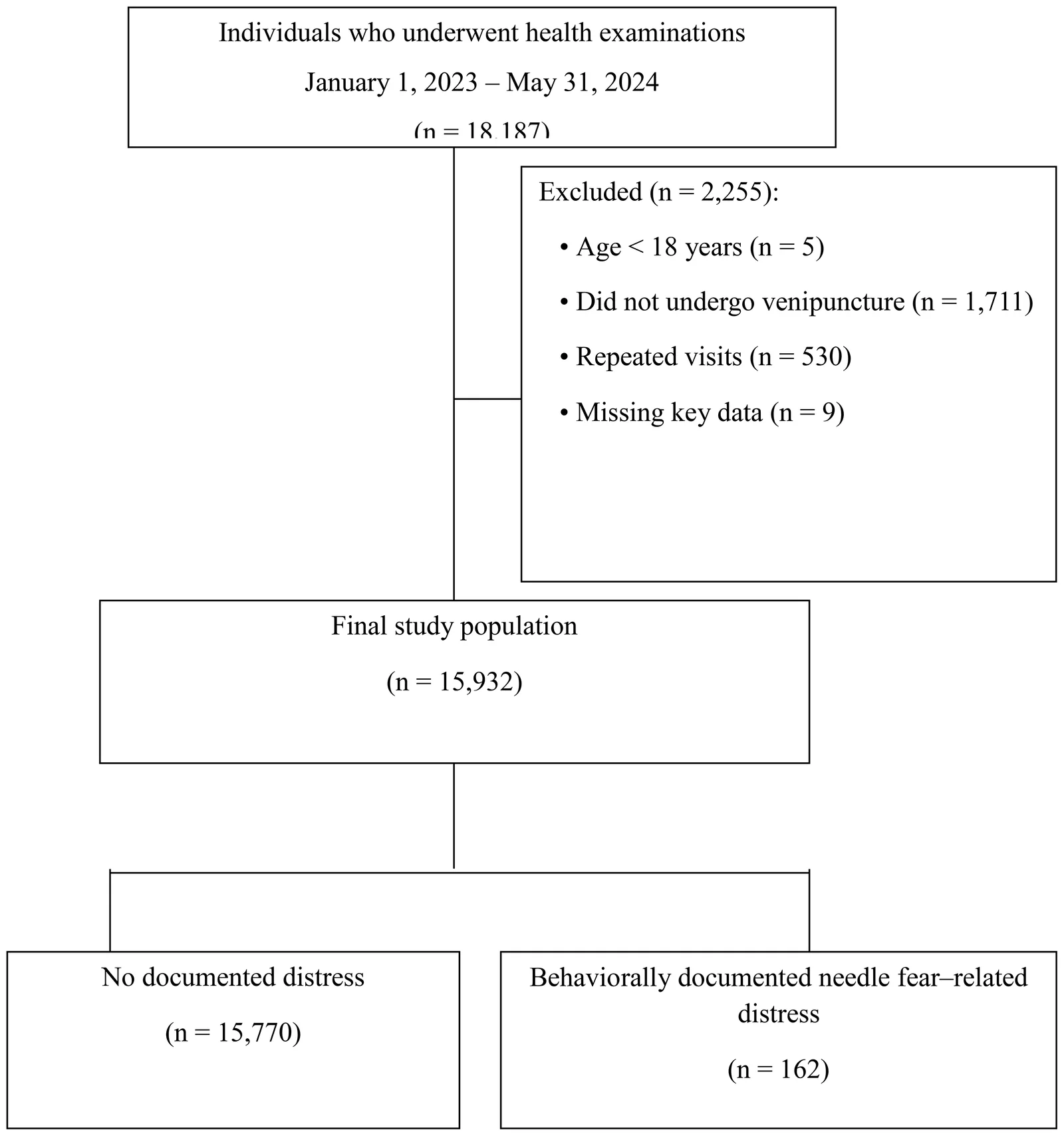
Flow diagram of participant selection. A total of 18,187 individuals underwent health examinations during the study period. After applying exclusion criteria, 15,932 unique adults who received venipuncture were included in the final analysis. Among them, 162 individuals (1.02%) exhibited behaviorally documented needle fear–related distress.

The study size was determined by the number of individuals available in the institutional database during the study period. The study population included adults who underwent routine health examinations at the study center between January 1, 2023, and May 31, 2024. A total of 18,187 individuals were initially identified from the institutional health examination database. Individuals younger than 18 years of age were excluded (n = 5), as the study focused exclusively on adult examinees. Participants who did not undergo venipuncture as part of their examination were also excluded (n = 1,711), since blood-drawing–related variables were essential for evaluating needle fear–associated responses. To ensure independence of observations, repeated visits by the same individual during the study period were removed, retaining only the first examination record for each participant (n = 530). Records with missing or incomplete information on key variables, including demographic characteristics and blood-drawing behavioral records, were also excluded (n = 9). After applying all exclusion criteria, a total of 15,932 unique individuals were included in the final analysis (**Figure 1**). No *a priori* sample size calculation was performed because all eligible records available during the predefined study period were included.

### 2.3 Data collection and variables

All data were retrieved from the institutional health examination database and were provided by the clinical informatics division of the participating medical center. Prior to analysis, all records were fully de-identified to ensure participant confidentiality. The dataset included multiple domains of health examination information. Demographic and behavioral characteristics included age, sex, cigarette smoking status, alcohol consumption, and betel nut use. Medical history variables included self-reported histories of previously diagnosed dyslipidemia, hypertension, and diabetes mellitus, as well as psychosomatic conditions such as insomnia, anxiety, or depression. Vital signs recorded during the health examination included body mass index, body temperature, systolic blood pressure, diastolic blood pressure, and heart rate. Laboratory parameters were obtained through routine venous blood sampling. Hematologic indices included white blood cell count, red blood cell count, hematocrit, hemoglobin concentration, and platelet count. Hepatic and renal function were assessed using serum albumin, alanine aminotransferase, aspartate aminotransferase, blood urea nitrogen, creatinine, and estimated glomerular filtration rate (eGFR). Electrolyte measurements included serum sodium, potassium, and calcium levels. Metabolic markers included fasting plasma glucose, glycated hemoglobin, total cholesterol, triglycerides, high-density lipoprotein cholesterol, low-density lipoprotein cholesterol, and uric acid levels. Thyroid function was assessed by measurement of thyroid-stimulating hormone concentration. Gastrointestinal findings were extracted from reports of esophagogastroduodenoscopy (EGD) and colonoscopy conducted as part of routine health examinations.

### 2.4 Assessment of needle fear–related indicators

During venipuncture procedures, trained nursing staff routinely documented examinee behavior and physiological responses in standardized nursing records. These observations included examinee cooperation during the procedure, visible anxiety responses (such as agitation, hesitation, or avoidance behavior), and physiological manifestations of distress, including dizziness, pallor, diaphoresis, or near-syncope. Because formal psychiatric evaluation for specific phobias is not performed during routine health examinations, these behavioral and physiological observations were used as pragmatic proxy indicators of needle fear for analytical purposes. Individuals who exhibited at least one marked anxiety response or sign of physiological distress during venipuncture were categorized as exhibiting needle fear–related indicators. The classification was based on routine, non-standardized nursing documentation rather than a validated observational instrument. No inter-rater reliability assessment was available.

### 2.5 Statistical analysis

Descriptive statistics were used to summarize demographic characteristics, health examination variables, and blood-drawing behavioral records. Continuous variables were presented as means with standard deviations, whereas categorical variables were reported as frequencies and percentages. Continuous variables were analyzed as continuous measures without categorization unless otherwise specified. Bivariate analyses were conducted to examine associations between needle fear–related indicators and health examination items. Continuous variables with non-normal distributions were analyzed using the Mann–Whitney U test, while associations between categorical variables were evaluated using the chi-square test or Fisher’s exact test, as appropriate. Variables showing statistically significant associations in the univariable analyses were further evaluated based on clinical relevance, representativeness, and potential collinearity. Sixteen variables were selected for inclusion in the multivariable logistic regression model to achieve a parsimonious and clinically interpretable model. Because EGD and colonoscopy exhibited severe multicollinearity, with both variables showing a tolerance of 0.01 and a VIF of 138.16, only colonoscopy was retained in the final model based on its greater potential to influence physiological responses during venipuncture. Results were reported as odds ratio (OR) with corresponding 95% confidence intervals (CI). Participants with missing data on key variables were excluded from the analysis. A two-sided p value < 0.05 was considered statistically significant. All statistical analyses were performed using SPSS version 26 (IBM Corp., Armonk, NY, USA).

### 2.6 Ethical considerations

All data were fully de-identified prior to analysis to protect participant confidentiality. The study protocol was reviewed and approved by the institutional review board of the participating medical center on September 2, 2025 (approval number: CE25697A). Because the study involved retrospective analysis of de-identified administrative and clinical data, the requirement for informed consent was waived in accordance with institutional and national ethical guidelines.

## 3 Results

Among the 15,932 individuals included in the final analysis, 162 (1.02%) had documented behavioral or physiological distress during venipuncture. The baseline demographic and clinical characteristics of the study population are presented in **Table 1**. Individuals with needle fear were significantly younger than those without needle fear (mean age: 42.06 ± 11.71 vs. 49.04 ± 12.95 years, p < 0.001). The prevalence of hypertension and diabetes mellitus was significantly lower in the needle fear group (hypertension: 9.26% vs. 17.5%, p = 0.006; diabetes mellitus: 1.85% vs. 7.69%, p = 0.005). No significant differences were observed between groups in sex distribution, psychosomatic disorders (insomnia, anxiety, or depression), or lifestyle behaviors including smoking, betel nut use, and alcohol consumption.

**Table 1:** Baseline characteristics of the study population according to needle fear status.

Characteristic	Total (n=15932)	Needle fear		*P* value
		No (n=15770)	Yes (n=162)	
Age (years)	48.96 ± 12.96	49.04 ± 12.95	42.06 ± 11.71	<0.001
Sex				0.295
Female, n (%)	7733 (48.54%)	7661 (48.58%)	72 (44.44%)	
Male, n (%)	8199 (51.46%)	8109 (51.42%)	90 (55.56%)	
Chronic diseases				
Dyslipidemia, n (%)	3290 (20.65%)	3264 (20.70%)	26 (16.05%)	0.146
Hypertension, n (%)	2775 (17.42%)	2760 (17.5%)	15 (9.26%)	0.006
Diabetes mellitus, n (%)	1216 (7.63%)	1213 (7.69%)	3 (1.85%)	0.005
Psychosomatic disorder				
Insomnia, n (%)	1519 (9.53%)	1501 (9.52%)	18 (11.11%)	0.492
Anxiety, n (%)	752 (4.72%)	741 (4.70%)	11 (6.79%)	0.212
Depression, n (%)	402 (2.52%)	399 (2.53%)	3 (1.85%)	0.801
Lifestyle				
Smoking, n (%)	1977 (12.50%)	1952 (12.46%)	25 (15.53%)	0.242
Betel nut use, n (%)	195 (1.23%)	192 (1.23%)	3 (1.86%)	0.454
Alcohol consumption, n (%)	7431 (46.95%)	7346 (46.89%)	85 (52.80%)	0.135

Comparisons of vital signs, endoscopic findings, and laboratory parameters are summarized in **Table 2**. Participants with needle fear had a significantly lower body mass index (23.36 ± 4.05 vs. 24.19 ± 3.84 kg/m², p = 0.005) and systolic blood pressure (114.95 ± 16.35 vs. 121.09 ± 17.38 mmHg, p < 0.001). The proportion of participants undergoing EGD and colonoscopy was significantly higher in the needle fear group (85.19% for each procedure) compared with the non-needle fear group (EGD: 62.63%; colonoscopy: 62.54%; both p < 0.001).

**Table 2:** Clinical, endoscopic, and laboratory characteristics according to needle fear status.

Characteristic	Total (n=15932)	Needle fear		*P* value
		No (n=15770)	Yes (n=162)	
Body mass index (kg/m²)	24.19 ± 3.84	24.19 ± 3.84	23.36 ± 4.05	0.005
Vital sign				
Body temperature (°C)	36.16 ± 0.45	36.16 ± 0.45	36.13 ± 0.45	0.394
Systolic blood pressure (mmHg)	121.02 ± 17.38	121.09 ± 17.38	114.95 ± 16.35	<0.001
Diastolic blood pressure (mmHg)	74.51 ± 11.61	74.52 ± 11.61	73.63 ± 11.75	0.159
Heart rate (beats/min)	71.71 ± 11.05	71.7 ± 11.05	72.75 ± 11.14	0.196
Esophagogastroduodenoscopy, n (%)	10015 (62.86%)	9877 (62.63%)	138 (85.19%)	<0.001
Colonoscopy, n (%)	10000 (62.77%)	9862 (62.54%)	138 (85.19%)	<0.001
Laboratory data				
White blood cell count (×10³/µL)	5.68 ± 1.68	5.67 ± 1.68	6.19 ± 1.57	<0.001
Red blood cell count (×10^6^/µL)	4.82 ± 0.56	4.81 ± 0.56	4.90 ± 0.56	0.019
Hematocrit (%)	42.42 ± 4.12	42.42 ± 4.12	43.05 ± 4.31	0.023
Hemoglobin (g/dL)	14.16 ± 1.59	14.15 ± 1.58	14.44 ± 1.69	0.009
Platelet count (×10³/µL)	256.67 ± 61.96	256.53 ± 61.98	270.3 ± 59.59	0.002
Serum albumin (g/dL)	4.57 ± 0.26	4.57 ± 0.26	4.66 ± 0.28	<0.001
Aspartate aminotransferase (U/L)	24.16 ± 14.13	24.17 ± 14.17	23.33 ± 9.74	0.293
Alanine aminotransferase (U/L)	26.28 ± 22.97	26.28 ± 22.99	26.01 ± 20.25	0.386
Blood urea nitrogen (mg/dL)	11.12 ± 3.92	11.13 ± 3.93	10.03 ± 2.76	<0.001
Serum creatinine (mg/dL)	0.85 ± 0.30	0.85 ± 0.30	0.85 ± 0.20	0.667
Estimated glomerular filtration rate (mL/min/1.73 m²)	91.06 ± 18.85	91.02 ± 18.85	94.47 ± 17.93	0.017
Serum sodium (mmol/L)	140.67 ± 2.23	140.68 ± 2.23	139.97 ± 2.29	<0.001
Serum potassium (mmol/L)	4.00 ± 0.33	4.00 ± 0.33	3.91 ± 0.32	<0.001
Serum calcium (mg/dL)	9.27 ± 0.36	9.27 ± 0.36	9.32 ± 0.34	0.057
Hemoglobin A1c (%)	5.66 ± 0.72	5.66 ± 0.72	5.50 ± 0.62	<0.001
Fasting blood glucose (mg/dL)	94.57 ± 19.12	94.63 ± 19.16	89.12 ± 14.33	<0.001
Total cholesterol (mg/dL)	197.73 ± 38.53	197.65 ± 38.46	205.12 ± 43.83	0.086
Serum triglycerides (mg/dL)	105.85 ± 78.03	105.79 ± 77.87	111.33 ± 92.27	0.251
High-density lipoprotein cholesterol (mg/dL)	55.63 ± 15.78	55.63 ± 15.76	54.95 ± 17.81	0.196
Low-density lipoprotein cholesterol (mg/dL)	124.77 ± 35.37	124.7 ± 35.32	131.8 ± 39.49	0.043
Serum uric acid (mg/dL)	5.79 ± 1.46	5.79 ± 1.46	5.92 ± 1.61	0.254
Thyroid-stimulating hormone (µIU/mL)	1.85 ± 2.25	1.85 ± 2.25	1.91 ± 1.20	0.214

Several hematological parameters differed significantly between the two groups. Individuals with needle fear had a higher white blood cell count compared with those without needle fear (6.19 ± 1.57 vs. 5.67 ± 1.68, p < 0.001). They also exhibited higher red blood cell count, hematocrit, hemoglobin concentration, and platelet count (all p < 0.05).

Several biochemical markers also showed significant differences. Serum albumin levels and eGFR were higher in the needle fear group (eGFR: 94.47 ± 17.93 vs. 91.02 ± 18.85, p = 0.017). Conversely, individuals with needle fear had lower levels of blood urea nitrogen, sodium, potassium, hemoglobin A1c, and fasting blood glucose (all p < 0.001). Among lipid profile parameters, only low-density lipoprotein cholesterol was significantly higher in the needle fear group (131.8 ± 39.49 vs. 124.7 ± 35.32 mg/dL, p = 0.043). No statistically significant differences were observed in liver enzyme levels, body temperature, heart rate, calcium concentration, thyroid-stimulating hormone levels, total cholesterol, triglycerides, high-density lipoprotein cholesterol, or serum uric acid.

The results of the univariate and multivariate logistic regression analyses are shown in **Table 3**. The multivariable model was adjusted for age, and selected representative clinical variables to account for potential confounding effects. Age remained independently associated with needle fear (adjusted OR = 0.96, 95% CI: 0.94–0.98, p < 0.001). Systolic blood pressure was also independently associated with needle fear (adjusted OR = 0.98, 95% CI: 0.97–0.99, p = 0.005). Participants who underwent colonoscopy had significantly higher odds of needle fear (adjusted OR = 3.49, 95% CI: 2.01–6.06, p < 0.001). White blood cell count also remained statistically significant in the adjusted model (adjusted OR = 1.11, 95% CI: 1.05─1.18, p < 0.001), although the magnitude of the effect was small.

**Table 3:** Factors associated with documented venipuncture-related distress.

Variable	Univariate		Multivariable	
	OR (95 % CI)	*P* value	OR (95 % CI)	*P* value
Age	0.96 (0.95 ─ 0.97)	<0.001	0.96 (0.94 ─ 0.98)	<0.001
Male vs Female	1.18 (0.86 ─ 1.61)	0.295		
Chronic diseases				
Dyslipidemia	0.73 (0.48 ─ 1.12)	0.148		
Hypertension	0.48 (0.28 ─ 0.82)	0.007	1.13 (0.61 ─ 2.08)	0.702
Diabetes mellitus	0.23 (0.07 ─ 0.71)	0.011	0.42 (0.12 ─ 1.47)	0.173
Psychosomatic disorder				
Insomnia	1.19 (0.73 ─ 1.95)	0.493		
Anxiety	1.48 (0.80 ─ 2.74)	0.215		
Depression	0.73 (0.23 ─ 2.29)	0.586		
Lifestyle				
Smoking	1.29 (0.84 ─ 1.98)	0.244		
Betel nut use	1.53 (0.48 ─ 4.83)	0.470		
Alcohol consumption	1.27 (0.93 ─ 1.73)	0.136		
Body mass index (kg/m²)	0.94 (0.90 ─ 0.98)	0.006	0.96 (0.91 ─ 1.02)	0.159
Vital sign				
Body temperature (°C)	0.86 (0.61 ─ 1.21)	0.397		
Systolic blood pressure (mmHg)	0.98 (0.97 ─ 0.99)	<0.001	0.98 (0.97 ─ 0.99)	0.005
Diastolic blood pressure (mmHg)	0.99 (0.98 ─ 1.01)	0.334		
Heart rate (beats/min)	1.01 (0.99 ─ 1.02)	0.227		
Esophagogastroduodenoscopy	3.43 (2.22 ─ 5.30)	<0.001		
Colonoscopy	3.44 (2.23 ─ 5.32)	<0.001	3.49 (2.01 ─ 6.06)	<0.001
Laboratory data				
White blood cell count (×10³/µL)	1.09 (1.04 ─ 1.14)	<0.001	1.11 (1.05 ─ 1.18)	<0.001
Red blood cell count (×10^6^/µL)	1.31 (1.01 ─ 1.70)	0.044	1.23 (0.90 ─ 1.69)	0.200
Hematocrit (%)	1.04 (1.00 ─ 1.08)	0.052		
Hemoglobin (g/dL)	1.13 (1.02 ─ 1.25)	0.022		
Platelet count (×10³/µL)	1.003 (1.001 ─ 1.01)	0.004	1.00 (1.00 ─ 1.00)	0.480
Serum albumin (g/dL)	3.66 (2.04 ─ 6.59)	<0.001	1.17 (0.59 ─ 2.34)	0.649
Aspartate aminotransferase (U/L)	0.99 (0.98 ─ 1.01)	0.442		
Alanine aminotransferase (U/L)	1.00 (0.99 ─ 1.01)	0.881		
Blood urea nitrogen (mg/dL)	0.91 (0.86 ─ 0.95)	<0.001	1.03 (0.98 ─ 1.08)	0.220
Serum creatinine (mg/dL)	0.98 (0.57 ─ 1.69)	0.934		
Estimated glomerular filtration rate (mL/min/1.73 m²)	1.01 (1.001 ─ 1.02)	0.020		
Serum sodium (mmol/L)	0.89 (0.84 ─ 0.94)	<0.001	0.96 (0.89 ─ 1.04)	0.372
Serum potassium (mmol/L)	0.43 (0.26 ─ 0.70)	0.001	0.60 (0.34 ─ 1.08)	0.089
Serum calcium (mg/dL)	1.44 (0.96 ─ 2.17)	0.078		
Hemoglobin A1c (%)	0.58 (0.41 ─ 0.82)	0.002	1.01 (0.75 ─ 1.35)	0.955
Fasting blood glucose (mg/dL)	0.97 (0.96 ─ 0.99)	<0.001		
Total cholesterol (mg/dL)	1.005 (1.001 ─ 1.01)	0.014	1.00 (0.99 ─ 1.01)	0.942
Serum triglycerides (mg/dL)	1.00 (1.00 ─ 1.00)	0.365		
High-density lipoprotein cholesterol (mg/dL)	1.00 (0.99 ─ 1.01)	0.584		
Low-density lipoprotein cholesterol (mg/dL)	1.01 (1.001 ─ 1.01)	0.011	1.00 (0.99 ─ 1.02)	0.396
Serum uric acid (mg/dL)	1.06 (0.96 ─ 1.18)	0.237		
Thyroid-stimulating hormone (µIU/mL)	1.01 (0.95 ─ 1.06)	0.771		

## 4 Discussion

### 4.1 Statement of principal findings

This large retrospective study identified needle fear–related behaviors in approximately 1.02% of adults undergoing routine health examinations. This prevalence is consistent with previous estimates of clinically significant needle phobia reported in systematic reviews, which typically range from approximately 1% to 4% of the adult population (1). Unlike many previous investigations in which needle fear has been assessed primarily through self-reported questionnaires or psychometric scales, needle fear–related behaviors in the present study were documented through real-time behavioral observations during venipuncture in a routine health examination setting (3).

Participants exhibiting needle fear–related behaviors were significantly younger than those without such behaviors. This finding is consistent with previous research indicating that needle-related fear is more prevalent in younger populations and tends to decline with age (3, 16). The lower prevalence of hypertension and diabetes mellitus observed in the needle fear group may partly reflect the younger age distribution of this population. In addition, avoidance of healthcare procedures associated with needle fear has been described in prior studies and may influence patterns of healthcare utilization among affected individuals (4).

Physiological distress observed during venipuncture among individuals with needle fear is consistent with established models of the vasovagal response associated with blood-injection-injury phobia (7, 9, 10). This subtype of specific phobia has been characterized by a distinctive diphasic autonomic response involving an initial sympathetic activation followed by parasympathetic overcompensation, which may result in hypotension, dizziness, presyncope, or syncope during exposure to needle-related stimuli (7, 9). Such physiological reactions may contribute to the behavioral distress observed during venipuncture procedures in routine healthcare settings (19).

### 4.2 Strengths and weaknesses in relation to other studies

The prevalence observed in the present study is lower than rates of self-reported needle fear reported in population surveys but is consistent with estimates derived from stricter definitions of clinically significant needle phobia (1, 13–15). Differences across studies have frequently been attributed to variations in measurement approaches, particularly the reliance on self-reported fear scales rather than direct behavioral observation (3, 13, 16).

Previous investigations have highlighted the importance of validated psychometric instruments for evaluating the cognitive and emotional dimensions of needle fear (12, 27, 28). These tools are designed to quantify subjective fear responses and cognitive aspects of phobia that may not be observable during medical procedures. In contrast, the present study captured observable behavioral distress documented during routine clinical practice. This observational approach may therefore provide ecologically valid insight into real-world responses to medical procedures, although subclinical or internally experienced fear may not be fully detected using behavioral documentation alone (12).

### 4.3 Implications for clinicians and policymakers

Needle phobia has been recognized as a clinically meaningful barrier to preventive healthcare and chronic disease management (4, 16, 21). Avoidance of needle-related procedures has been associated with delayed medical care, reduced participation in vaccination programs, and inadequate monitoring of chronic diseases (1, 4). Such avoidance behaviors may ultimately compromise preventive healthcare engagement and long-term disease control.

Improved recognition of needle phobia–related behaviors during routine health examinations may therefore facilitate early identification of individuals experiencing substantial procedural distress. Implementation of structured screening approaches for needle-related anxiety, together with evidence-based interventions such as applied muscle tension techniques and exposure-based psychological therapies, has been recommended to improve tolerance of needle-related medical procedures (21–23). A recent systematic review further synthesized evidence on exposure-based treatments for fear and heightened reactivity across various medical procedures, highlighting their potential clinical relevance while also identifying the need for further research on treatment implementation and outcomes (29). Emerging interventions, including virtual reality–assisted exposure therapy and physiological monitoring systems designed to anticipate vasovagal reactions, have also demonstrated promising preliminary findings and warrant further investigation (24, 25).

### 4.4 Strengths and weaknesses of the study

Several strengths of the present study should be acknowledged. The analysis was conducted using a large sample of individuals undergoing routine health examinations, allowing needle fear–related behaviors to be evaluated within a real-world clinical setting. In addition, behavioral observations recorded during venipuncture were integrated with comprehensive demographic, clinical, and laboratory data obtained during the health examination process.

Nevertheless, several limitations should also be considered. First, needle phobia was not diagnosed using standardized psychiatric diagnostic criteria but was inferred from behavioral observations documented during venipuncture procedures. Misclassification was therefore possible. In particular, participants experiencing substantial internal fear without visible behavioral or physiological manifestations may have been classified as not having distress. Second, the retrospective cross-sectional design limits the ability to establish causal relationships between clinical variables and needle fear–related behaviors. Third, the study was conducted at a single tertiary medical center, which may limit the generalizability of the findings to other healthcare settings or populations.

### 4.5 Unanswered questions and future research

Future research should incorporate validated psychometric instruments to improve diagnostic precision in the assessment of needle fear and phobia (12, 28). Prospective longitudinal studies may further clarify developmental trajectories of needle fear and associated avoidance behaviors across the lifespan (3, 16). Additional investigation is also needed to optimize management strategies for populations particularly vulnerable to needle-related distress, including individuals with neurodevelopmental disorders and other conditions associated with heightened procedural anxiety (17, 18).

## 5 Conclusion

In this large retrospective study of individuals undergoing routine health examinations, needle fear–related behaviors were identified in approximately 1% of adult examinees. Participants exhibiting needle fear–related responses were significantly younger and demonstrated clinical characteristics consistent with previously described physiological reactions associated with blood-injection-injury phobia. The findings highlight that needle-related distress may occur during routine health screening and may influence healthcare experiences during blood-drawing procedures. Improved recognition of needle fear–related behaviors may facilitate early identification of affected individuals and support the implementation of appropriate strategies to reduce procedural distress in preventive healthcare environments.

## Data Availability

The raw data supporting the conclusions of this article will be made available by the authors, without undue reservation.
